# Permeation of Polymethoxyflavones into the Mouse Brain and Their Effect on MK-801-Induced Locomotive Hyperactivity

**DOI:** 10.3390/ijms18030489

**Published:** 2017-02-24

**Authors:** Satoshi Okuyama, Kohei Miyazaki, Rie Yamada, Yoshiaki Amakura, Morio Yoshimura, Atsushi Sawamoto, Mitsunari Nakajima, Yoshiko Furukawa

**Affiliations:** 1 Department of Pharmaceutical Pharmacology, College of Pharmaceutical Sciences, Matsuyama University, 4-2 Bunkyo-cho, Matsuyama, Ehime 790-8578, Japan; sokuyama@cc.matsuyama-u.ac.jp (S.O.); mu.yakuri.002@gmail.com (K.M.); 46140018@cc.matsuyama-u.ac.jp (A.S.); mnakajim@cc.matsuyama-u.ac.jp (M.N.); 2 Department of Pharmacognosy, College of Pharmaceutical Sciences, Matsuyama University, 4-2 Bunkyo-cho, Matsuyama, Ehime 790-8578, Japan; 46150023@cc.matsuyama-u.ac.jp (R.Y.); amakura@cc.matsuyama-u.ac.jp (Y.A.); myoshimu@cc.matsuyama-u.ac.jp (M.Y.)

**Keywords:** polymethoxyflavone, heptamethoxyflavone, nobiletin, tangeretin, natsudaidain, blood-brain-barrier, locomotive hyperactivity, ERK1/2, MK-801

## Abstract

Accumulating data have indicated that citrus polymethoxyflavones (PMFs) have the ability to affect brain function. In the present study, we showed that 3,5,6,7,8,3′,4′-heptamethoxy- flavone (HMF) given intraperitoneally to mice was immediately detected in the brain and that the permeability of the brain tissues to it was significantly higher than that of other citrus PMFs (nobiletin, tangeretin, and natsudaidain). The permeation of these PMFs into the brain well correlated with their abilities to suppress MK-801-induced locomotive hyperactivity, suggesting that HMF had the ability to act directly in the brain. We also obtained data suggesting that the suppressive effect of HMF on MK-801-induced locomotive hyperactivity was mediated by phosphorylation of extracellular signal-regulated kinases 1/2 (ERK1/2) in the hippocampus.

## 1. Introduction

**Figure 1:**
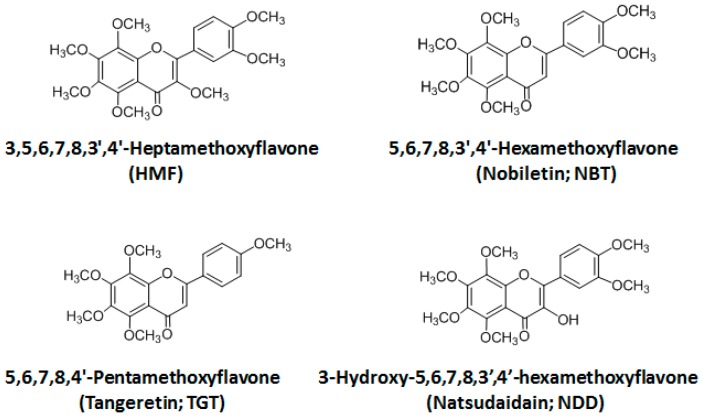
Chemical structures of 3,5,6,7,8,3′,4′-heptamethoxy-flavone (HMF), nobiletin (NBT), tangeretin (TGT), and natsudaidain (NDD).

Citrus species are a rich source of polymethoxyflavones (PMFs) and their hydroxylated derivatives, which have a broad spectrum of biological activity including anti-cancer, anti-inflammatory, and anti-oxidant effects [1]. Recent studies have revealed that PMFs are able to exert powerful actions not only in peripheral tissues but also in the brain [2]. Specifically, the anti-dementia action of nobiletin (5,6,7,8,3′,4′-hexamethoxyflavone; NBT) has been vigorously studied [3,4]. Pharmacokinetic study revealed that NBT given intraperitoneally (i.p.) or orally (p.o.) was delivered to the brain tissues [5,6]. On the other hand, we have accumulated findings that peripherally administered 3,5,6,7,8,3′,4′-heptamethoxyflavone (HMF) has the ability to affect brain function: (1) the subcutaneous (s.c.) administration of HMF has a neuroprotective effect in the ischemic mouse brain [7,8], which might be mediated by the induction of brain-derived neurotrophic factor (BDNF) synthesis; (2) s.c. administration of HMF improves depression symptoms of mice [9], the improvement of which might also be mediated by the induction of BDNF synthesis; (3) s.c. administration of HMF attenuates lipopolysaccharide (LPS)-induced inflammation in the mouse brain [10]; (4) s.c. administration of HMF improves MK-801-induced hyperactive behavior [11]. However, we have not yet investigated whether HMF acts directly or indirectly in the brain. Namely, we have not yet investigated whether HMF is able to pass into the brain or not. Consequently, the first aim of the present study was to develop methods to analyze serum/brain levels of HMF and to determine whether HMF could penetrate the blood–brain barrier (BBB). As a result, we successfully revealed that HMF could indeed pass through this BBB. Then, we compared the BBB-permeability toward HMF with that of a series of other citrus PMFs. In addition to HMF, which has 7 methoxy groups at 3,5,6,7,8,3′,4′ positions, this series included NBT with 6 methoxy groups at 5,6,7,8,3′,4′ positions, tangeretin (TGT) with 5 methoxy groups at 5,6,7,8,4′ positions, and natsudaidain (3-hydroxy-5,6,7,8,3′,4′-hexamethoxyflavone; NDD) with a 3-hydroxy group instead of the 3-methoxy group of HMF (Figure 1). As the route of administration of PMFs in the present study, we adopted i.p. injection instead of s.c. injection, because i.p. injection was shown to be a convenient alternative to s.c. injection [12]. The dose of PMFs was 50 mg/kg/day (50 mg is equivalent to 116 μmol for HMF, 124 μmol for NBT, 134 μmol for TGT, and 120 μmol for NDD), because our previous studies [7,8,9,10,11] and other studies [3,4,5,6,13] showed that this dose might be adequate to examine their brain function and their BBB permeability.

The second aim of the present study was to examine whether the differences in the BBB permeability among four PMFs influenced their ability to affect brain function. As the “brain function”, we selected here their suppressive effect on MK-801-induced locomotive hyperactivity [11].

A previous report showed that consolidation of learning likely requires learning-elicited activation of extracellular signal-regulated kinases 1/2 (ERK1/2), which are components of the mitogen-activated protein kinase (MAPK) signaling cascade, via NMDA receptors [14]. We previously found that HMF has the ability to activate (that is to say phosphorylate) ERK1/2 in vitro [15] and in vivo [9]. The last aim of the present study was to determine whether the suppressive effect of HMF on MK-801-induced locomotive hyperactivity was mediated by phosphorylation of ERK1/2 or not.

## 2. Results and Discussion

### 2.1. Determination of Serum/Brain Levels of 3,5,6,7,8,3′,4′-Heptamethoxyflavone after Intraperitoneal Administration

Analytical HPLC methods used to determine the contents of NBT and TGT in serum or brain tissues have been already developed [5,6,13]. Based on this method, we developed the HPLC/UV method in order to analyze auraptene (AUR), a citrus coumarine derivative [16]. In the present study, we adopted this method to analyze the serum/brain levels of citrus PMFs.

**Figure 2:**
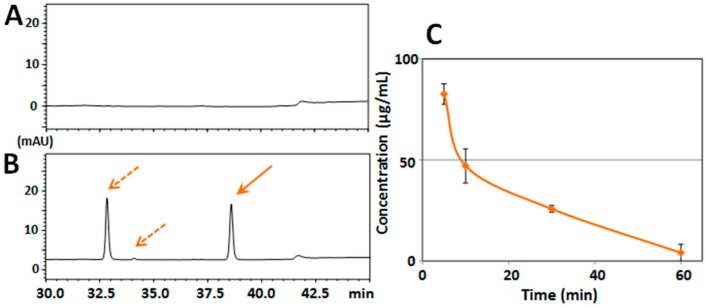
(**A**) HPLC chromatograms of serum from vehicle-treated mouse; and (**B**) HPLC chromatograms of serum from mice treated intraperitoneally (i.p.) with HMF. Solid arrow is HMF and dashed arrows are putative metabolites of HMF; (**C**) Time-course of the serum concentration profiles of HMF following i.p. administration. Values are the means ± SEM.

At first, we evaluated the mouse serum/brain levels of HMF after its single-dose administration. For Experiment I, mice (*n* = 3 at each time point) were i.p. administered HMF (50 mg/kg), and tissue samples (serum and brain) were collected at 5, 10, 30, and 60 min after the administration. A typical chromatogram of a non-treated serum sample revealed no interfering peaks at the retention time of HMF (Figure 2A), and that of a serum sample from an HMF-treated mouse (after 5 min) revealed a sharp peak of HMF (solid arrow in Figure 2B) together with another large sharp peak and a small peak (dashed arrows in Figure 2B). As the prior analysis by HPLC revealed that the purity of HMF was more than 98% (data not shown), the two peaks given by dashed arrows were not impure substances of HMF but its metabolites. The mean concentration–time profile of HMF in serum (Figure 2C) shows that the content of HMF in the serum reached its highest level of data (82.6 ± 5.1 μg/mL) at 5 min after the administration and steeply declined to 47.1 ± 8.4 μg/mL at 10 min, to 25.8 ± 1.8 μg/mL at 30 min, and to 4.1 ± 4.2 μg/mL at 60 min, indicating that HMF might have been quickly absorbed.

**Figure 3:**
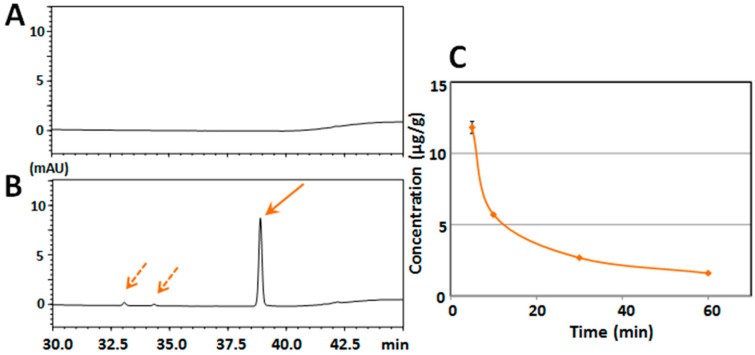
(**A**) HPLC chromatogram of brain tissue from vehicle-treated mouse; and (**B**) HPLC chromatogram of brain tissue from mice treated i.p. with HMF. Solid arrow is HMF and dashed arrows are putative metabolites of HMF; (**C**) Time-course of the brain concentration profiles of HMF following i.p. administration. Values are the means ± SEM.

Figure 3A shows that there were no interfering peaks at the retention time of HMF in the typical chromatogram of the brain sample from a non-treated mouse. Figure 3B shows that there was a sharp peak of HMF (solid arrow) and two other small peaks (dashed arrows) in the typical chromatogram of the brain sample from an HMF-treated mouse (after 5 min). The results of Figure 3A,B indicate that HMF was present in the brain together with a small quantity of metabolites. 

The mean concentration–time profile of HMF in the brain tissue (Figure 3C) shows that the content of HMF in the brain reached its highest level (11.81 ± 0.43 μg/g of brain tissue) at 5 min after the administration, followed by a steep decline to 5.69 ± 0.07 μg/g at 10 min, 2.67 ± 0.05 μg/g at 30 min, and 1.59 ± 0.06 μg/g at 60 min, indicating that the HMF in the circulating blood may have quickly penetrated into the brain and that the main metabolite in serum might have scarcely passed through the BBB, because it might be a hydrophilic form.

### 2.2. Structure–Activity Relationship of Permeability of the Blood–Brain Barrier to Polymethoxyflavones

We next evaluated the mouse serum/brain levels of NBT, TGT, and NDD after i.p. administration in order to compare their ability to permeate the BBB with that of HMF. Tissue samples (serum and brain) of mice were generated following administration of each PMF (50 mg/kg).

**Figure 4:**
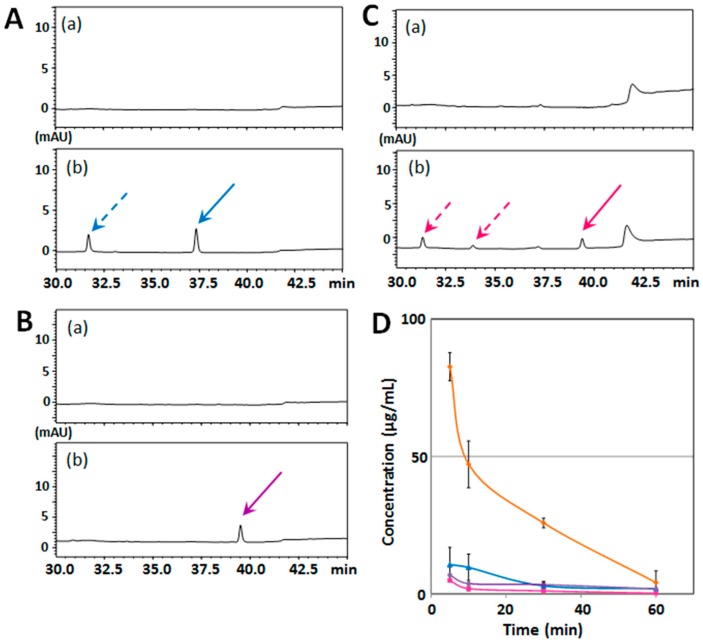
HPLC chromatograms of serum from vehicle-treated mouse (**a**) and after (**b**) i.p. injection with NBT (**A**), TGT (**B**), or NDD (**C**). Solid arrows are each PMFs and dashed arrows are putative metabolites of PMFs. (**D**) Time-course of serum concentration profiles of HMF (orange line), NBT (blue line), TGT (purple line), and NDD (red line) following i.p. administration. Values are the means ± SEM.

Figure 4A–C show sets of typical chromatograms of serum from a mouse before (a); and after (b) injection with NBT (A), TGT (B), or NDD (C). The findings that the purity of NBT and NDD was more than 98% (data not shown) suggested that NBT (solid arrow in Figure 3A) and NDD (solid arrow in Figure 3C) was present with its metabolite(s) in serum (dashed arrow(s) in Figure 3A,C). The mean concentration–time profiles of each PMF in serum (Figure 4D) shows that the serum levels of NBT (blue line), TGT (purple line), and NDD (red line) were substantially lower than that of HMF (orange line). 

The serum content of NBT was calculated to be 10.6 ± 6.3 μg/mL at 5 min and 2.9 ± 1.6 μg/mL at 30 min after its administration, which agreed with the data of Saigusa et al. [5], who reported that the mouse serum level of NBT at 3 min after i.p. injection (at a dose of 50 mg/kg) was 7.18 μg/mL. The serum content of TGT was 6.8 ± 0.4 μg/mL at 5 min and 3.4 ± 0.9 μg/mL at 30 min after its administration, corresponding with the data of [13], the authors of which reported that the rat serum level of TGT at 30 min after i.p. injection (at a dose of 50 mg/kg) was 4.5 μg/mL. The serum content of NDD was the lowest among the 4 PMFs, i.e., 4.8 ± 0.5 μg/mL at 5 min and 1.1 ± 0.3 μg/mL at 30 min. 

**Figure 5:**
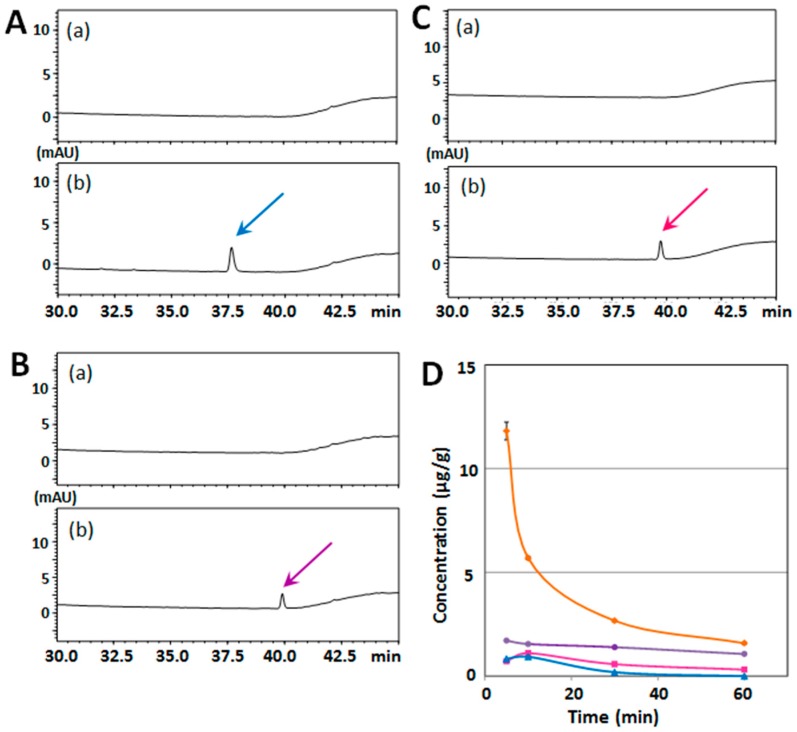
HPLC chromatograms of brain tissue from vehicle-treated mouse (**a**) and after (**b**) i.p. injection with NBT (**A**), TGT (**B**), or NDD (**C**). (**D**) Time-course of the brain concentration profiles of HMF (orange line), NBT (blue line), TGT (purple line), and NDD (red line) following i.p. administration. Colored arrows indicate respective PMFs. Values are the means ± SEM.

Figure 5A–C show the same type of data for the brain samples. The results suggested that the metabolite(s) of NBT and NDD in serum might have scarcely passed through the BBB, possibly because these metabolite(s) were hydrophilic form(s). The mean concentration–time profiles of each PMF in brain tissues (Figure 5D) shows that the brain levels of NBT (blue line), TGT (purple line), and NDD (red line) were substantially lower than that level of HMF (orange line). The NBT brain content was 0.836 ± 0.00001 μg/g at 5 min, 0.943 ± 0.00001 μg/g at 10 min, and 0.192 ± 0.00001 μg/g at 30 min, which was lower than the data reported by [5], who indicated that the mouse brain level of NBT at 5 min after i.p. injection (at a dose of 50 mg/kg) was 22 μg/g. The reason(s) for this discrepancy is (are) unknown. The highest content of TGT in brain was 1.72 ± 0.0055 μg/g at 5 min; and that of NDD, 1.11 ± 0.00683 μg/g at 10 min.

These results indicate that the number and position of methoxy groups of citrus PMFs might be important factors for their absorption into serum and subsequently into the brain.

### 2.3. Structure–Activity Relationship of the Suppressive Effect of PMFs on MK-801-Induced Locomotive Hyperactivity

It is well known that MK-801, an NMDA receptor antagonist, has the ability to induce a variety of symptoms in a dose-dependent manner, i.e., a cognitive deficit at a low dose, locomotor hyperactivity at a moderate dose [11], and immobility and deficits in social interaction at a high dose [17]. The locomotive hyperactivity induced by MK-801 is generally used as an indicator of schizophrenia-like behavior [18,19]. We previously showed that HMF has the suppressive effect on the MK-801-increased total distance traveled (an indicator of general locomotive activity) in the open field [11]. We thus used this effect of HMF (suppressive effect on MK-801-induced locomotive hyperactivity in open field) in order to determine the relationship between the structures of citrus PMFs and the extent of their ability as neurotrophic agents.

In Experiment II, there were 6 experimental groups (*n* = 7 at group): a non-treated group (control group), a MK-801-injected group (MK group), and 4 MK-801-injected plus PMF-treated groups, namely, a MK-801 and HMF-treated group (MK + HMF group), a MK-801 and NBT-treated group (MK + NBT group), a MK-801 and TGT-treated group (MK + TGT group), and a MK-801 and NDD-treated group (MK + NDD group). The mice of these MK + PMF groups received daily s.c. injections of each PMF (50 mg/kg/day) for 7 days. In the same way, mice of the control group and the MK group received daily s.c. injections of the vehicle (1:1 solution of dimethyl sulfoxide (DMSO)/polyethylene glycol (PEG) 300) for 7 days. Thirty minutes after the last sample administration on the 7th day, mice of the MK group and MK + PMF groups were i.p. injected with MK-801 (0.2 mg/kg). Mice of the control group were i.p. injected with the vehicle (saline). Thirty minutes after the MK-801 injection (namely, 1 h after the last administration of each PMF), the locomotive activity of each mouse was evaluated by using the open field test.

**Figure 6:**
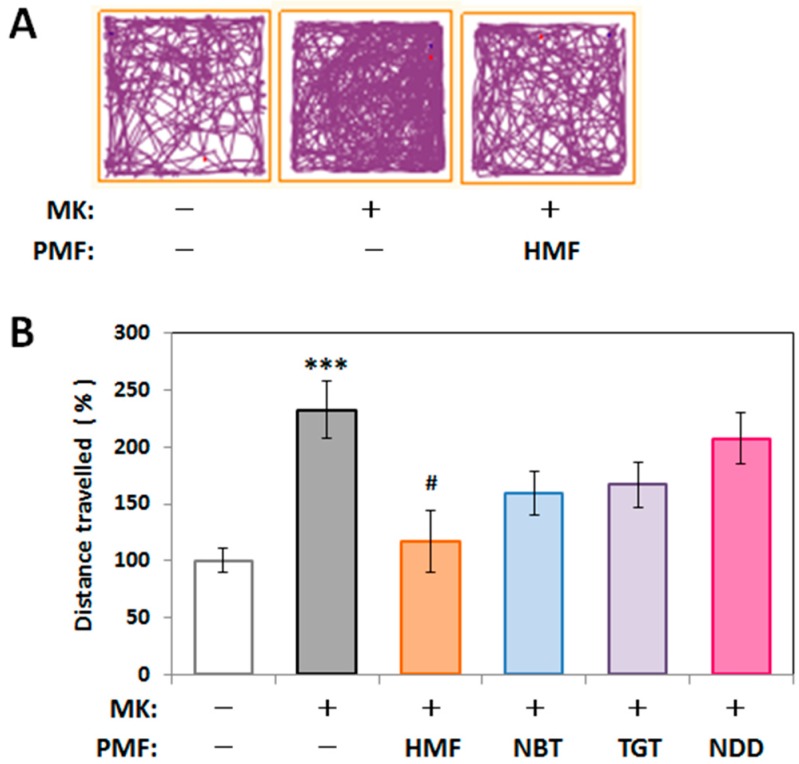
Effect of PMFs on the spontaneous locomotive behavior of MK-801-injected mice in the open field test. (**A**) Representative traces during 10 min; (**B**) Total distance traveled in the open field test during the 10-min period. Values (% control) are the means ± SEM. *** *p* < 0.001 vs. control group. ^#^ *p* < 0.05 vs. MK group.

Figure 6A shows representative traces in the open field test for the control group, the MK group, and the MK + HMF group. Figure 6B shows that the total distance traveled (%) during the experimental period by the MK group (233% ± 25.0%) was significantly (* *p* < 0.001) higher than that of the control group (100% ± 10.5%; measured value was 45.73 ± 4.82 m). A significant decrease in the total distance traveled of the MK + HMF group (117% ± 27.3%) compared with the MK group was observed (one-way ANOVA *p* < 0.05, F_(4,29)_ = 3.816, Tukey’s post hoc test: ^#^ *p* < 0.05), in accordance with our previous results [11]. NBT and TGT have a tendency to suppress the MK-801-induced increase in locomotive activity (159% ± 19.0% and 167% ± 20.2%, respectively), but there were no statistical significant differences compared to the MK-801-injected group. Meanwhile, NDD treatment had no obvious effect (207% ± 22.7%).

These results suggested that the protective effect of citrus PMFs on MK-801-induced locomotive hyperactivity in the open field test might be influenced by their ability to permeate the BBB (Figure 5).

### 2.4. Mechanism of Inhibitory Effect of HMF on MK-801-Induced Locomotive Hyperactivity

A previous report showed that learning-elicited activation of ERK1/2 via NMDA receptors is necessary for consolidation of learning [14]. We previously found that HMF has the ability to activate (phosphorylate) ERK1/2 [9,15]. The last aim of the study was thus to determine whether the protective effect of HMF on MK-801-induced locomotive hyperactivity was mediated by phosphorylation of ERK1/2, and we did so by using U0126, an inhibitor of MAPK/ERK kinase (MEK). 

In Experiment III, there were 6 experimental groups (*n* = 4 at group); a non-treated group (control group), a U0126-treated group (U0126 group), a MK-801-injected group (MK group), a MK-801-injected and U0126-treated group (MK + U0126 group), a MK-801-injected plus HMF-treated group (MK + HMF group), and a MK-801-injected plus U0126 + HMF group (MK + HMF + U0126 group). For the mice of the HMF-treated groups, HMF at a dose of 50 mg/kg was s.c. injected. For those of the MK-injected groups, MK-801 was i.p. injected at a dose of 0.2 mg/kg 30 min after the last HMF treatment. For the mice of the U0126-injected groups, U0126 was i.p. injected at a dose of 0.4 mg/kg 30 min before the HMF administration (namely, 1 h before the MK-801 injection). Thirty minutes after the MK-801 injection, (namely, 1 h after the last administration of HMF, and 1.5 h after the injection of U0126), the locomotive activity of mice was evaluated by use of the open field test.

**Figure 7:**
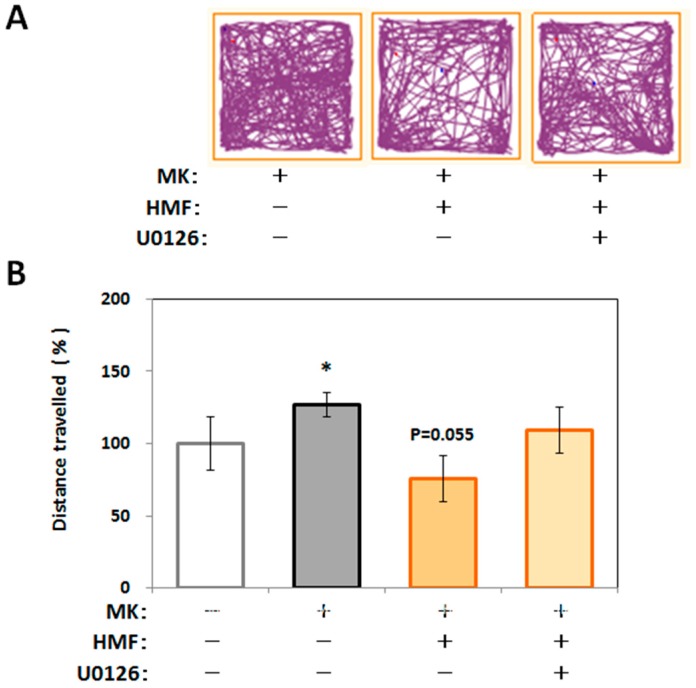
Effect of U0126 on the spontaneous locomotive behavior of MK-801 and/or HMF-injected mice in the open field test. (**A**) Representative traces during 10 min; (**B**) Total distance traveled in the open field test during the 10-min period. Values (% control) are the means ± SEM. * *p* < 0.05 vs. control group. *p* = 0.055 vs. MK group.

Figure 7A shows representative traces in the open field test for the MK group, the MK + HMF group, and the MK + HMF + U0126 group. Figure 7B shows that the locomotive activity (% of the total distance traveled) of the mice in the MK group (127.4% ± 8.5%) was significantly (* *p* < 0.05) higher compared with that of the control group (100% ± 6.3%; measured value was 34.2 ± 6.3 m). One-way ANOVA revealed no statistical significant effect on the total distance traveled among the three groups (*p* > 0.05, F_(2,19)_ = 3.206, Tukey’s post hoc test: MK + HMF group vs. MK group, *p* = 0.055; MK + HMF + U0126 group vs. MK group, *p* = 0.634). It scarcely needs to be said that the treatment with U0126 itself did not affect the locomotive activity (96.9% ± 9.7%) and that the ability of MK-801 to heighten the locomotive activity was not affected by U0126 (127.4% ± 22.2%).

**Figure 8:**
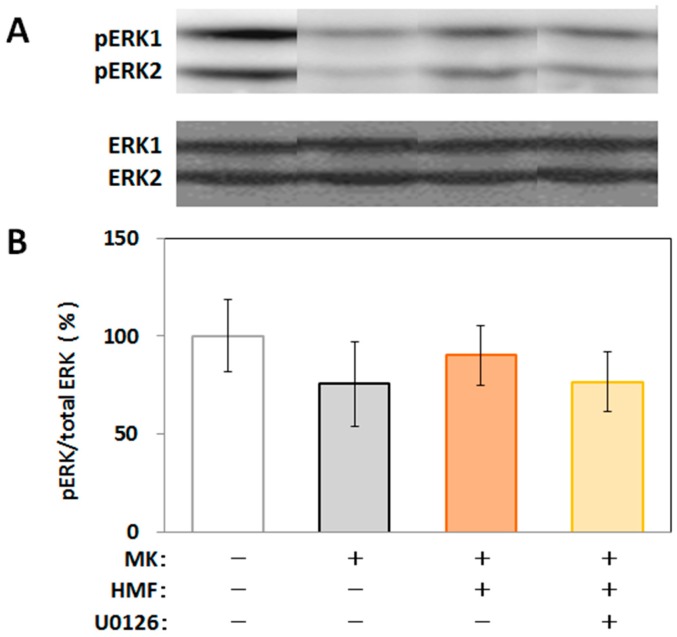
Western blot analysis of phosphorylation of ERK (pERK)1/2 in the U0126 and/or MK-801-treated mouse hippocampus. (**A**) Representative band patterns of pERKs and ERKs; (**B**) Quantitative analysis of the pERK/ERK ratios by use of Image J software. Values are the means ± SEM.

We then applied the brain tissues to Western blot analysis to examine the level of phosphorylated ERK1/2 in the hippocampus. As shown in Figure 8, MK-801 tended to suppress the level of phosphorylated ERK1/2 in hippocampus, HMF tended to suppress this effect of MK-801, and U0126 tended to suppress this effect of HMF. The reason why there was no statistical significance among the groups might be that the phosphorylation of signal transduction factors is temporary and that we could not detect any significant differences under the present experimental conditions. 

The important results of the present study were that HMF given i.p. could penetrate the BBB and that the ability of HMF to permeate the BBB was substantially higher than that of NBT, TGT, and NDD. As a result, the effect on the level of brain function (as determined by the suppressive effect on MK-801-induced-hyperlocomotion) of HMF was the highest among the PMFs examined here (HMF, NBT, TGT, and NDD). As PMFs in circulating blood might quickly penetrate into brain (Figure 2, Figure 3, Figure 4 and Figure 5), we are now investigating the intestinal absorption of PMFs. Furthermore, we are also now investigating the BBB permeation of HMF when it is p.o. injected, in order to use HMF as a therapeutic agent in the future. It would be also the important problem to investigate whether the metabolites of PMFs have any function in the brain.

The brain content of HMF at 5 min (12 μg/g) was calculated to correspond to about 28 μM, and this amount might be effective in the brain, because HMF tended to affect the phosphorylation of ERK1/2 (Figure 2 and Figure 8). The previous studies by Saigusa et al. [5] indicated that the NBT content in the brain (~55 μM) is high enough to stimulate cAMP/PKA/ERK/CREB signaling.

## 3. Materials and Methods

### 3.1. Chemicals and Reagents

HMF, NBT, and NDD were kindly provided by Ushio ChemiX Corporation (Omaezaki, Japan). HPLC revealed that their purity was more than 98%. TGT was prepared from commercial orange oil (Wako, Osaka, Japan) as in the case of HMF previously described [15]. HPLC revealed that its purity was more than 95%. MK-801 ((+)-MK-801 hydrogen maleate) and U0126 were purchased from Sigma-Aldrich (St. Louis, MO, USA) and Wako, respectively.

### 3.2. Determination of PMFs via HPLC/UV

We applied a method similar to that for measuring brain levels of citrus auraptene [16] to analyze the serum/brain levels of citrus PMFs. Each standard stock solution of PMFs was prepared by dissolving them in methanol. Stock solutions were diluted to prepare a calibration curve with linearity in the range of 0.1–10 μg/mL. HPLC analysis was performed by using a Shimadzu Prominence system (Shimadzu, Kyoto, Japan). Reversed-phase (RP) HPLC conditions were as follows: column, L-column ODS (5 μm, 150 mm × 2.1 mm i.d.; Chemicals Evaluation and Research Institute, Tokyo, Japan); mobile phase, solvent A was 5% acetic acid, and solvent B was acetonitrile gradient: (0→30 min (0%→50% B in A), 30→35 min (50%→85% B in A), 35→40 min (85% B in A), 40→50 min (85%→100% B in A)); injection volume, 2 μL; column temperature, 40 °C; flow rate, 0.3 mL/min; detection, 340 nm for HMF, 330 nm for NBT, 320 nm for TGT, and 360 nm for NDD. The limit of quantification (LOQ) was 0.100 μg/mL for HMF, 0.039 μg/mL for NBT, 0.074 μg/mL for TGT, and 0.037 μg/mL for NDD. Each value was taken as means ± SEM of 3 analysis.

### 3.3. Animals

Male ICR strain mice from Japan SLC (Hamamatsu, Japan) were used at 7 weeks of age for experiment I, at 8 weeks of age for experiment II, and at 9 weeks of age for experiment III. Mice were housed in a room maintained at a constant temperature of 23 ± 1 °C with 12-h light/dark cycle (lights on 8:00–20:00). Mice were given food and water ad libitum for the duration of the study. All animal care and experimental procedures were performed in accordance with the Guidelines for Animal Experimentation and the Animal Care and Use Committee of Matsuyama University, and the experiments were implemented by the approved protocol (No. 9002, 2 September 2009).

### 3.4. Treatment with Reagents and Blood/Tissue Preparation

For i.p. administration of PMFs in Experiment I, PMFs were dissolved in 1:9 solution of DMSO/saline. In order to collect blood, mice were deeply anesthetized with diethyl ether, and approximately 1 mL of blood was collected by cardiac puncture from the right ventricle. After that, the mice were rapidly perfused with heparinized phosphate-buffered saline (PBS) through the left ventricle, after which their brains were excised. The blood was left on ice to clot, and serum was obtained as the supernatant after centrifugation of 800× *g* at 4 °C for 10 min. For s.c. administration of PMFs in Experiments II and III, PMFs were dissolved in a 1:1 solution of DMSO/PEG 300. MK-801 and U0126 was dissolved in saline and 1% DMSO in saline, respectively. In order to prepare hippocampus tissues in Experiments III, mice under anesthesia with diethyl ether were perfused with ice-cold PBS through the left ventricle, and their brains were then excised, as was previously described in detail [7]. These serum/brain samples were stored at −80 °C until used for analysis.

### 3.5. Sample Preparation for HPLC/UV

For the HPLC analysis of serum, 50 μL of serum sample were combined with 25 μL of methanol, vortexed for 5 min, and centrifuged at 14,000× *g* at 4 °C for 10 min. For the HPLC analysis of brain, approximately 250 mg of brain was sonicated with an equal weight of H_2_O for 30 s by use of a Sonicator 3000 (Misonix Inc., Farmingdale, NY, USA). The homogenate (100 μL) was added to 150 μL of methanol and then vortexed for 5 min. Supernatants were recovered after centrifugation at 14,000× *g* at 4 °C for 10 min and used as the samples.

### 3.6. Open Field Test

Each mouse was placed in the center of an open field apparatus (W70 cm × D70 cm × H50 cm), and free moving behavior was monitored by use of an ANY-maze Video Tracking System (Stoelting, Wood Dale, IL, USA). During the observation period for 10 min, the total distance was examined. Since it was reported that MK-801 treatment for 35 min results in the maximal activation of locomotion, which then gradually declines over the experimental period [18], we evaluated the locomotive activity of each mouse at 30 min after the MK-801 injection, as previously reported [11].

### 3.7. Western Blot Analysis

Tissue extracts were prepared as described before [7], and the equal amounts of protein (20 μg) were applied for immunoblot analysis (*n* = 4 for each group). The antibody against ERK1/2 (rabbit antibody against MAPK 1/2) as a primary antibody and the alkaline phospatase (AP)-linked anti-rabbit IgG (donkey) as a secondary antibody were obtained from Cell Signaling (Danvers, MA, USA) and Promega (Madison, WI, USA), respectively. Then, the activity of AP was visualized by using nitro blue tetrazolium/bromochloroindolyl phosphate color substrate.

Both the antibody against phosphorylated ERK1/2 (rabbit antibody against p 44/42 MAPK) as a primary antibody and the horseradish peroxidase (HRP)-linked anti-rabbit IgG (goat) as a secondary antibody were obtained from cell signaling. Then, the blots were developed by use of the chemiluminescence method with ECL-Prime (GE Healthcare, Chalfont St., UK).

### 3.8. Statistical Analysis

The data were expressed as means ± SEM. In Experiment II, significant differences were analyzed by using the unpaired *t*-test between the control and MK group to determine the validity of MK-801 treatment in the experiment, and then one-factor ANOVA followed by the Tukey’s Multiple Comparison Test among MK-treated five groups to determine the effects of PMFs (Prism 6; GraphPad Software, La Jolla, CA, USA). In Experiment III, significant differences were analyzed by using the unpaired *t*-test between the control and MK group to determine the validity of MK-801 treatment, and then one-factor ANOVA followed by Tukey’s Multiple Comparison Test among three groups (MK group, MK + HMF group and MK + HMF + U0126 group) to determine the effects of HMF and U0126 (Prism 6). The criterion for significance was *p* < 0.05 in all statistical evaluations.

## 4. Conclusions

The present study revealed that HMF i.p. injected could pass through the BBB and that this ability was considerably higher than that of the other citrus PMFs studied (NBT, TGT, and NDD). The ability of HMF to suppress MK-801-induced locomotive hyperactivity was the highest among these PMFs, which well correlated with their ability to permeate the brain. The present study also revealed that the suppressive effect of HMF on MK-801-induced locomotive hyperactivity was mediated by phosphorylation of ERK1/2 in the hippocampus.
